# HIF1A protein expression is correlated with clinical features in gastric cancer: an updated systematic review and meta-analysis

**DOI:** 10.1038/s41598-024-63019-6

**Published:** 2024-06-14

**Authors:** Seungyoon Nam, Yeeun Lee

**Affiliations:** 1 Department of Health Sciences and Technology, Gachon Advanced Institute for Health Sciences and Technology (GAIHST), Gachon University, Incheon, 21999 Republic of Korea; 2 Department of Genome Medicine and Science, AI Convergence Center for Medical Science, Gachon Institute of Genome Medicine and Science, Gachon University Gil Medical Center, Gachon University College of Medicine, 38-13, 3Beon-gil Dokjeom-ro, Namdong-gu, Incheon, 21565 Republic of Korea

**Keywords:** HIF1A, Gastric cancer, Meta-analysis, Immunohistochemistry, Gastric cancer, Cancer epidemiology, Cancer screening

## Abstract

To elucidate the correlation of HIF1A with clinicopathologic characteristics in patients with gastric cancer (GC), we conducted a systematic review and meta-analysis. We searched PubMed, Embase and Web of Science for studies on GC and HIF1A, covering studies published until January 31st, 2022. We calculated odds ratios (ORs) and 95% confidence intervals (CIs) for clinical characteristics based on high and low HIF1A protein levels. We used random-effects and fixed-effects meta-analysis methods to determine mean effect sizes of ORs and evaluated publication heterogeneity with τ^2^, I^2^, and Q values. Additionally, we generated funnel plots to inspect publication bias. Our meta-analysis included 20 publications with 3416 GC patients to estimate the association between high or low HIF1A expression and clinical characteristics. Positive HIF1A expression was significantly associated with T stage progression (OR: 2.46; 95% CI 1.81–3.36; P < 0.01), TNM stage progression (OR: 2.50; 95% CI 1.61–3.87; P < 0.01), lymph node metastasis (OR: 2.06; 95% CI 1.44–2.94; P < 0.01), undifferentiated status (OR: 1.83; 95% CI 1.45–2.32; P < 0.01), M stage progression (OR: 2.34; 95% CI 1.46–3.77; P < 0.01), Borrmann stage progression (OR: 1.48; 95% CI 1.02–2.15; P = 0.04), larger tumor size (OR: 1.27; 95% CI 1.06–1.52; P < 0.01), vascular invasion (OR: 1.94; 95% CI 1.38–2.72; P < 0.01), and higher vascular endothelial growth factor (VEGF) protein expression (OR: 2.61; 95% CI 1.79–3.80; P < 0.01) in our meta-analysis. GC Patients highly expressing HIF1A protein might be prone to tumor progression, poorly differentiated GC cell types, and a high VEGF expression.

## Introduction

There were 768,793 gastric cancer (GC)-related deaths and 1,089,103 new cases reported globally in 2020^1^. Compared with other areas, China, Japan, and Korea have a greater occurrence of GC. However, we lack effective GC therapies and biomarkers^2–4^.

GC progression is also associated with a hypoxic tumor microenvironment^5^. In hypoxia, hypoxia-inducible factor 1-alpha (HIF1A) is involved in the transcriptional regulation of genes related to angiogenesis, tumor invasion, cell survival, and glucose metabolism^6^. HIF1A is also involved in epithelial-to-mesenchymal transition (EMT) in GC via Snail^6^, promoting metastasis in GC^7^.

Few meta-analysis studies have been conducted for HIF1A in different cancers^8–10^. A study demonstrated the association of a *HIF1A* single nucleotide polymorphism (SNP) with the risk of various cancers, but GC was excluded from the meta-analysis^8^. In prostate cancer, HIF1A expression was significantly associated with the progression of the Gleason score^9^. Also, in a meta-analysis of GC^10^, positive HIF1A expression was correlated with poor survival rates, but only nine GC studies were evaluated.

However, the association between HIF1A protein levels and GC clinicopathologic features remains undefined. Thus, we need comprehensive evidence of a correlation between HIF1A and the clinicopathologic characteristics in GC.

In this systematic review and meta-analysis, we aimed to measure the statistical association between GC clinicopathological features and high or low HIF1A protein levels in GC patients. This meta-analysis summarizes earlier publications and examines whether HIF1A expression can predict clinicopathological traits including GC cell histology, GC progression, and invasion. By combining evidence from individual publications, this in-depth study shows the potential clinical correlations between protein levels of HIF1A and the severity of the clinicopathological features in patients with GC.

## Methods

### Literature search

**Figure 1:**
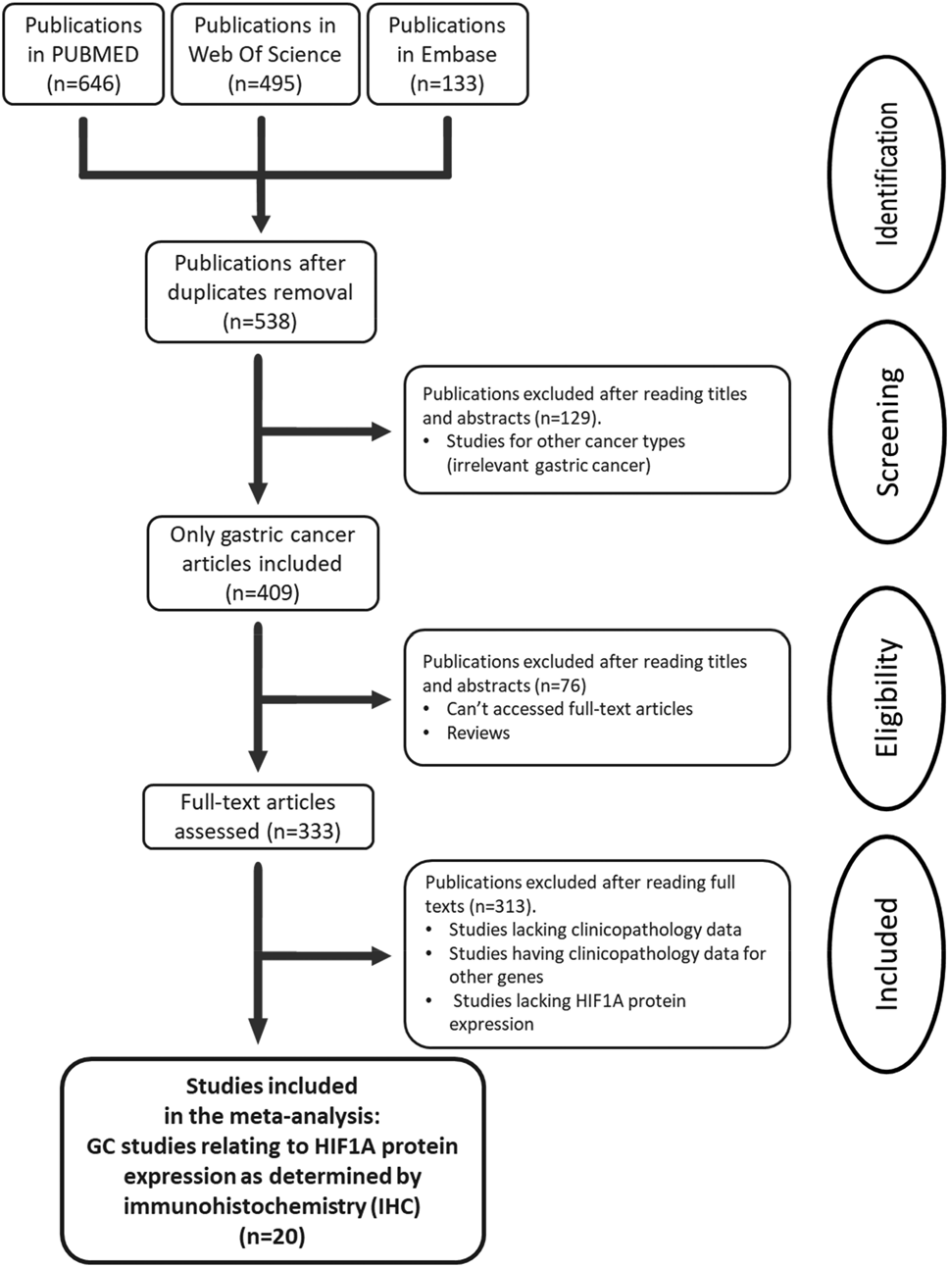
The systematic publication selection process.

Web of Science, Embase and PubMed were searched to identify appropriate publications, dated till Jan 31st, 2022, on HIF1A protein expression in GC for the meta-analysis. The search terms were “HIF1A,” “cancer,” “stomach,” “gastric”, and “expression” (Fig. 1).

### Selection criteria

The following criteria were considered to select the publications:

Inclusion criteria:

1. Case–control design studies in GC
2. GC studies on HIF1A protein levels using immunohistochemistry (IHC) staining
3. Studies having appropriate data for obtaining odds ratios (ORs) and their 95% confidence intervals (CIs).
4. Studies published till Jan 30th, 2022.

Exclusion criteria:

1. Studies on other genes
2. Reviews
3. Studies on other cancer types
4. Studies without clinical characteristics for HIF1A protein positive and negative expression
5. Non-English publications

### Study types

The included publications were retrospective case–control studies comparing high- and low-HIF1A protein levels in GC patients. The studies had clinical characteristics on gender, differentiation status, age, TNM stage progression, T stage progression, N stage progression, M stage progression, Borrmann types, tumor sizes, vascular endothelial growth factor (VEGF) status (negative or positive), vascular invasion, Lauren classification, and tumor sites.

### Data retrieval

The two authors (SN and YL) independently evaluated the literature that met the selection criteria. Publication year, authors, IHC staining for HIF1A, and clinical characteristics were described in Table 1 for the included studies^11–30^. The author (SN) reviewed and confirmed the data of the search results.

**Table 1:** Included publications for HIF1A immunohistochemistry (IHC) meta-analysis.

Publications (study names)	IHC score description	Antibodies	Clinical variables
Berlth et al.^11^ (Berlth, 2015)	HIF1A expression was classified by the proportion of primary tumor cells stained	Anti-HIF1A (mouse monoclonal; clone H1a67; Thermo Fisher Scientific)	TNM stage, T stage, M stage, N stage, tumor differentiation, gender, and Lauren classification
Hao et al.^12^ (Hao, 2019)	HIF1A expression was assessed by the proportion of cell nuclei positively stained	Anti-HIF1A (mouse monoclonal; ab16066; Abcam)	TNM stage, T stage, M stage, N stage, tumor differentiation, gender, and age
Isobe et al.^13^ (Isobe, 2013)	HIF1A expression was classified depending on the proportion of tumor cells stained	Anti-HIF1A (rabbit polyclonal; Santa Cruz Biotechnology)	T stage, M stage, N stage, VEGF expression, tumor differentiation, gender, and age
Jia et al.^14^ (Jia, 2013)	HIF1A score was rated by the percentage of positive tumor cells. Tumors scored as 3 + or 2 + were positive cases. tumors scored as 0 or 1 + were negative	Anti-HIF1A (mouse monoclonal; Santa Cruz Biotechnology),	TNM stage, N stage, tumor differentiation, tumor size, gender, and age
Mizokami et al.^15^ (Mizokami, 2006)	Staining intensity was scored as a coloring expression to obtain a HIF1A score	Anti-HIF1A (MAb clone H1a67; IgG2b; NB 100–105; Novus Biologicals)	N stage, VEGF expression, tumor differentiation, gender, and age
Wu et al.^16^ (Wu, 2017)	The score was classified into four grades, consdering the percentage of stained cells	Anti-HIF1A (rabbit; Abcam)	TNM stage, T stage, N stage, tumor differentiation, gender, tumor site, and age
Zhang et al.^17^ (Zhang, 2016)	HIF1A expression was assessed by the proportion of tumor cell nuclei positively stained	Anti-HIF1A (rabbit polyclonal; H206; Santa Cruz Biotechnology)	TNM stage, T stage, Vascular invasion, VEGF expression, Borrmann type, tumor differentiation, tumor size, gender, Lauren classification, and age
Lu et al.^18^ (Lu, 2013)	HIF1A score was defined by the percentage of positively stained cells	Anti-HIF1A (mouse monoclonal, MAB1935R and D)	TNM stage, T stage, M stage, N stage, Borrmann type, tumor differentiation, tumor size, gender, and age
Zhan et al.^19^ (Zhan, 2013)	HIF1A scoring standards were defined by the percentage of positive cells times the color intensity score, subsequently being averaged to obtain the final score	Anti-HIF1A (Boster Bio-Engineering)	TNM stage, T stage, N stage, VEGF expression, and Lauren classification
Yang et al.^20^ (Yang, 2015)	Staining intensity was scored as a coloring expression to obtain a HIF1A score	Anti-HIF1A (bs0737R; Biosynthesis Biotechnology)	TNM stage, T stage, M stage, N stage, Borrmann type, tumor differentiation, tumor size, gender, and age
Qiu et al.^21^ (Qiu, 2011)	HIF1A score was assessed by the tumor cell nuclei positively stained at the center and the invasive front of the tumor in each section	Anti-HIF1A (Ms-1164-P0; Neomarkers)	TNM stage, tumor differentiation, gender, tumor site, and age
Wang et al.^22^ (Wang, 2010)	The score was calculated by multiplying the intensity of immunoreactivity and the proportion of positively stained cells	Anti-HIF1A (mouse monoclonal; Chemicon)	TNM stage, T stage, N stage, gender, and age
Chen et al.^23^ (Chen, 2014)	The score was defined by the staining intensity times the proportion of positive cells	Anti-HIF1A (rabbit Monoclonal, 1:600, Epitomics)	T stage, N stage, Borrmann type, tumor differentiation, tumor size, gender, Lauren classification, and age
Zhang et al.^24^ (Zhang, 2017)	The score used the staining intensity and positive cell percentage	Anti-HIF1A (mouse monoclonal; Abcam)	TNM stage, T stage, vascular invasion, tumor differentiation, tumor size, gender, Lauren classification, and age
Griffiths et al.^25^ (Griffiths, 2007)	The HIF1A scoring system was classified by the nuclear staining intensity	Anti-HIF1A (mouse monoclonal; 610958; BD Biosciences)	TNM stage, T stage, M stage, N stage, tumor differentiation, and Lauren classification
Deng et al.^26^ (Deng, 2013)	The staining score was defined by the intensity of immunoreactivity proportion of positively stained cells	HIF1A primary antibody (Bioworld); anti-HIF1A (Epitomics)	TNM stage, T stage, N stage, tumor size, gender, and age
Han et al.^27^ (Han, 2019)	The HIF1A scoring system was classified by nuclear staining intensity	Anti-HIF1A (Abcam)	TNM stage, T stage, Borrmann type, tumor size, gender, Lauren classification, and age
Zhang et al.^28^ (Zhang, 2018)	HIF1A score was calculated by multiplying staining intensity by the proportion of positive cells	Anti-HIF1A (Abcam)	TNM stage, T stage, N stage, Borrmann type, tumor differentiation, tumor size, gender, Lauren classification, tumor site, and age
Kubo et al.^29^ (Kubo, 2016)	HIF-1A score was determined by nuclear staining in the tumor center and at the invasive front	Anti-HIF1A (rabbit monoclonal; EP1215Y; Epitomics)	TNM stage, T stage, N stage, vascular invasion, tumor differentiation, and gender
Jiang et al.^30^ (Jiang, 2019)	HIF1A score was calculated by multiplying the staining intensity by the proportion of positive cells	Anti-HIF1Α (rabbit antibody; Abcam)	TNM stage, T stage, N stage, tumor differentiation, gender, tumor site, and age

The 20 publications included to analyze HIF1A protein expression in this meta-analysis. In the studies, protein level was measured using immunohistochemistry (IHC). For each study, the HIF1A antibodies, IHC scoring schemes, and clinicopathological features were described. Study names for the meta-analysis are indicated in the first column.

### Assessment of quality

The evidence of quality was assessed according to Robinson et al.^31^ [Supplementary Table S1].

### Effect measures and assessment of heterogeneity

For the meta-analysis, the R package "meta"^32^ generated forest plots depicting ORs and 95% CIs from the selected studies. Random-effects or fixed-effects models (equivalently, common-effects model) assessed the pooled effect sizes of the ORs. The pooled effect size refers to the combined effect size estimates of the studies and is a crucial tool in determining the clinical association of high or low HIF1A protein levels in GC.

Heterogeneity was calculated using the R library "meta": between-study variance τ^2^ and Higgins’ I^2^, including Cochran’s Q-tests^32^. The fixed-effects models estimated pooled ORs when P ≥ 0.05 or I^2^ ≤ 50%, indicating the absence of heterogeneity^29^. Otherwise, the random-effects models generated pooled ORs^29,33^. The forest plots depicted clinical outcomes of GC patients who had high vs. low HIF1A protein expression.

Overexpression of HIF1A is crucial in cancer cell migration and proliferation^6^. However, the impact of HIF1A protein expression on clinical characteristics related to cancer cell migration and proliferation has not been thoroughly investigated. The proliferation and migration of cancer cells promote advanced cancer stages and cytological features. Therefore, we examined the clinical parameters, including TNM stage advancement and cancer cell differentiation status. We also inspected other clinical features (i.e., gender, age, T stage progression, N stage progression [lymph node metastasis], M stage progression, Borrmann types, tumor sizes, VEGF status, vascular invasion, Lauren classification, and tumor sites).

### Publication bias

Sensitivity analysis measured the effects of individual publications on the overall results by omitting one publication at a time. Subsequently, we examined the publication bias (standard error of OR vs. OR) using funnel plots and Egger’s tests. The absence of skewness and asymmetry in funnel plots often implies the absence of publication bias.

### Meta-regression

We employed a mixed-effects meta-regression model utilizing the R package “metafor” (version 4.4.0)^34^ to assess the impacts of covariates, age, and sex. Median ages and male-to-female ratios across individual studies were utilized as surrogate measures for age and sex, respectively. Meta-regression analysis was conducted for a given outcome when a covariate was present in a minimum of ten individual studies. We derived P-values to assess heterogeneity and to test the null hypothesis of no linear relationship between covariates and effect sizes in the meta-regression. Additionally, we presented bubble plots illustrating the associations of outcomes (i.e., ORs) with covariates.

## Results

### Literature search and included studies

PubMed, Embase and Web of Science searches reported 646, 133 and 495 studies, respectively. We discovered 538 publications after deleting duplicates and thoroughly inspecting the titles and abstracts. Then, 518 studies were discarded because of a lack of data and an unclear number of patients according to the selection criteria. Finally, 20 papers were chosen for the meta-analysis (Fig. 1).

There was a total of 3416 patients in the 20 articles, including 1784 HIF1A-positive and 1632 HIF1A-negative individuals with GC. IHC was used to evaluate HIF1A protein expression in patients with GC in these 20 studies (Table 1). The clinical features included gender, age, T stage progression, N stage progression, M stage progression, Borrmann types, tumor sizes, VEGF status, vascular invasion, Lauren classification, and tumor sites.

### Statistical correlations between HIF1A protein levels and clinical characteristics in GC

**Figure 2:**
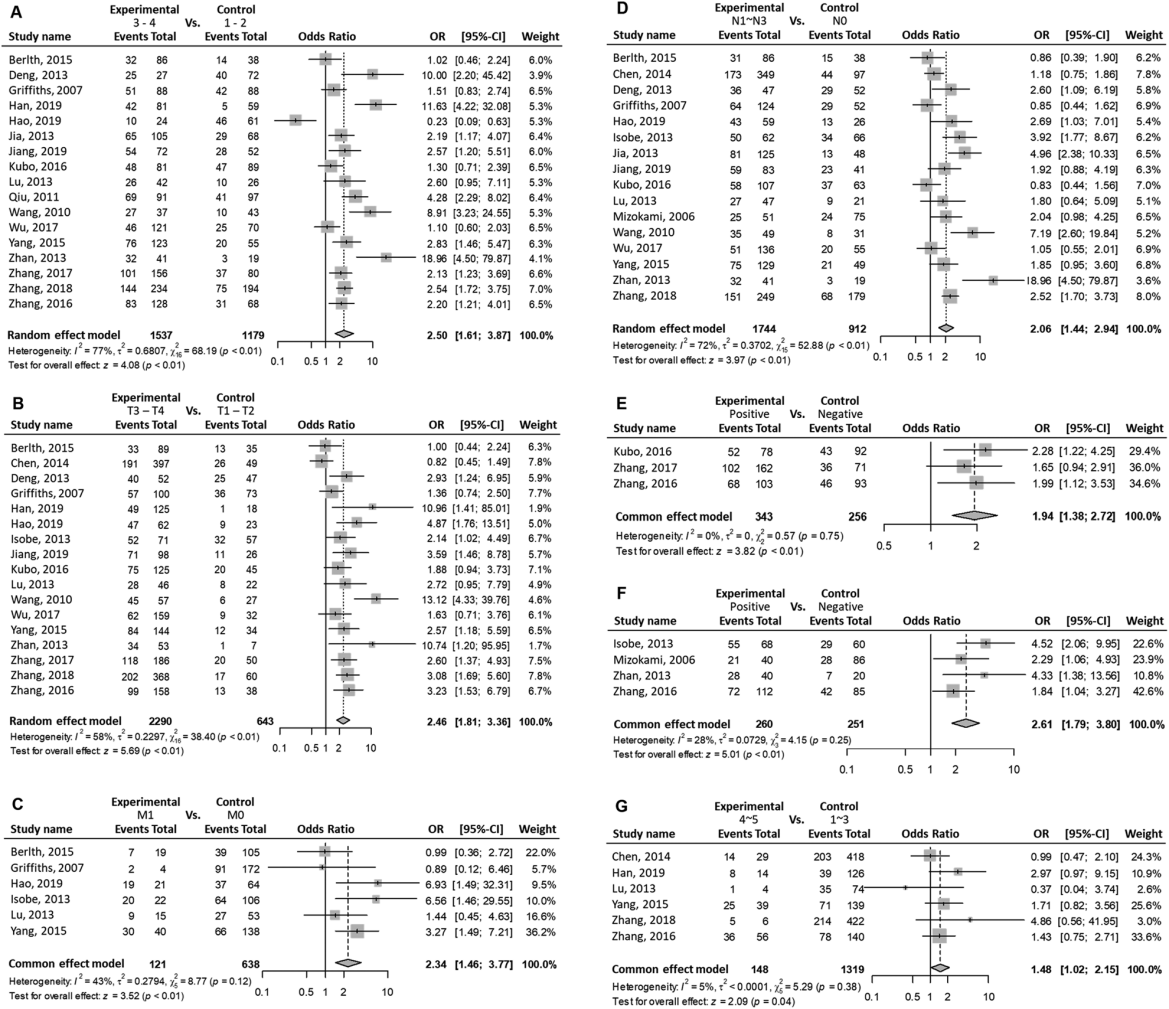

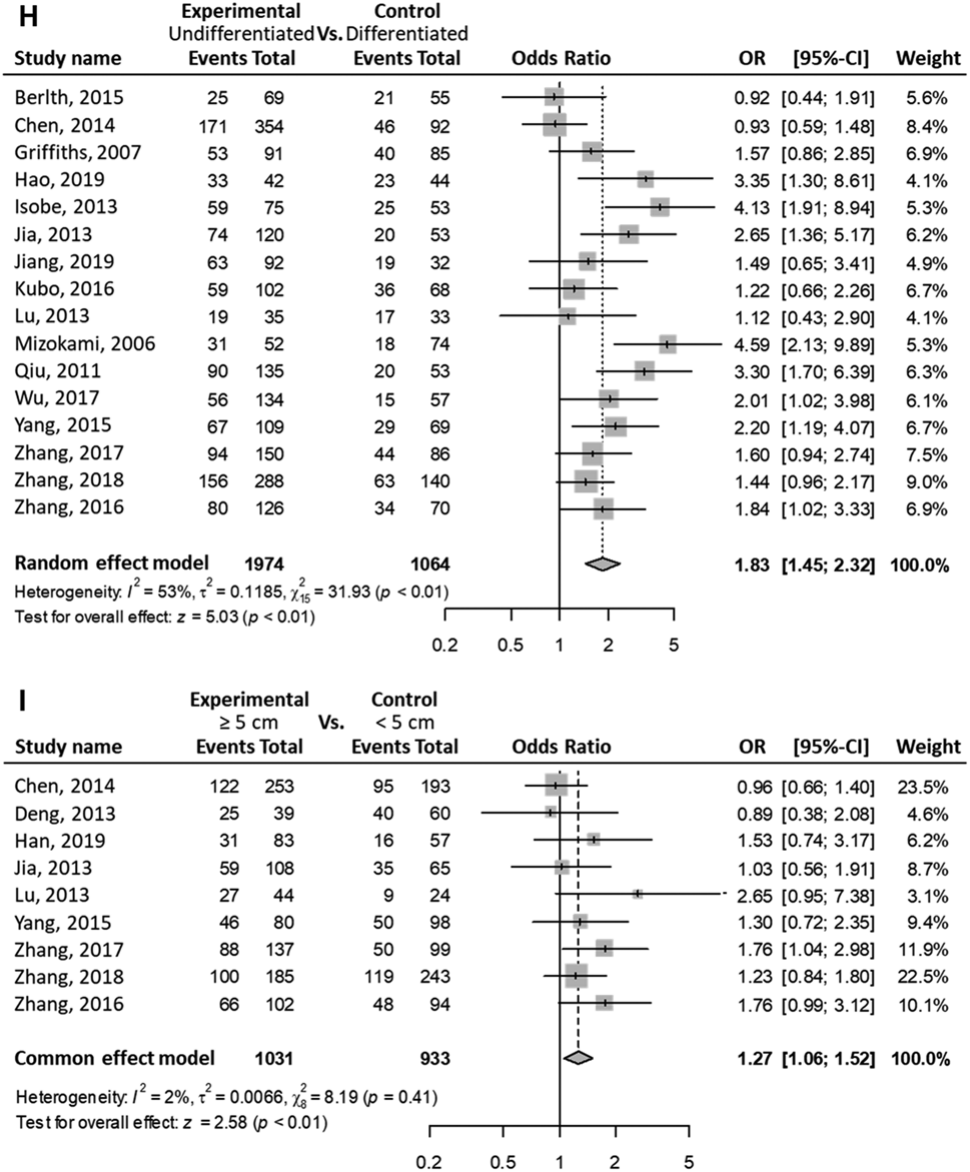
Meta-analysis on clinical characteristics. Events indicate high HIF1A expressing patients with GC (equivalently, HIF1A-positive patients with GC). Events suggest that patients with GC express high HIF1A protein levels. The first through sixth column lists: the study names, experimental group, control group, forest plots, odds ratios (ORs) of patients in the experimental group with high levels of HIF1A protein compared with those of control group, with 95% CI, and weight. One pooled effect size, OR, for high vs. low HIF1A levels in patients with GC is indicated with a clinicopathological feature. Between-study variance τ^2^, Higgins’ I^2^, and Cochran’s Q-tests were used to quantify heterogeneity. (**A**) TNM stages III–IV as experimental group vs. I–II as control group. In a group, events (i.e., high HIF1A protein-expressing GC patients) were obtained from a given study. The overall pooled effect estimate suggests that the OR of high or low HIF1A protein level between the two groups was > 1. High HIF1A-expressing patients are more in the experimental group (i.e., stages III–IV) than that in the control group (I–II). Positive HIF1A expression is associated with advanced TNM stages. (**B**) T stages T3–T4 vs. T1–T2. (**C**) M stages M1 vs. M0. (**D**) N stages N1–N3 vs. N0. (**E**) Positive vs. negative vascular invasion. (**F**) High (positive) vs. low (negative) VEGF expression. (**G**) Borrmann types 4–5 vs. 1–3. (**H**) Undifferentiated vs. differentiated differentiation statuses. (**I**) Tumor sizes ≥ 5 cm vs. < 5 cm.

Patients with positive HIF1A protein (equivalently, high) expression in GC were associated with the progression of the TNM stages (OR (III–IV vs. I–II) = 2.50; 95% CI 1.61–3.87; P < 0.01; random effects; Fig. 2A). Also, patients with high HIF1A expression in GC were significantly associated with the following clinical characteristics: progression of the T stages (OR (T3–T4 vs. T1–T2) = 2.46; 95% CI 1.81–3.36; P < 0.01; random effects; Fig. 2B); M stage progression (OR (M1 vs. M0) = 2.34; 95% CI 1.46–3.77; P < 0.01; fixed-effect; Fig. 2C); N stage progression (OR (N1–N3 vs. N0) = 2.06; 95% CI 1.44–2.94; P < 0.01; random effects; Fig. 2D); vascular invasion (OR (yes vs. no) = 1.94; 95% CI 1.38–2.72; P < 0.01; fixed-effect; Fig. 2E); VEGF protein positive expression in GC (OR (VEGF positive expression vs. VEGF negative expression) = 2.61; 95% CI 1.79–3.80; P < 0.01; fixed-effect; Fig. 2F); Borrmann stage progression (OR (stages 4–5 vs. 1–3) = 1.48; 95% CI 1.02–2.15; P = 0.04; fixed-effect; Fig. 2G); undifferentiated status (OR (undifferentiated vs. differentiated) = 1.83; 95% CI 1.45–2.32; P < 0.01; random-effect; Fig. 2H); and larger tumor size (OR (size ≥ 5 cm vs. size < 5 cm) = 1.27; 95% CI 1.06–1.52; P < 0.01; fixed-effect; Fig. 2I).

The other clinical features, including gender, age, tumor sites, and Lauren classification were not significantly associated with HIF1A expression in GC patients.

The Q-test P values for TNM stage progression, T stage progression, M stage progression, N stage progression, vascular invasion, VEGF positivity, Borrmann stage progression, differentiation status, and tumor size were < 0.01, < 0.01, 0.12, < 0.01, 0.75, 0.25, 0.38, < 0.01, and 0.41, respectively (Fig. 2). When the P value was < 0.05, the rejection of the null hypotheses of the same effect sizes in all studies indicated the effect sizes varied across studies.

### Robustness and publication bias

**Figure 3:**
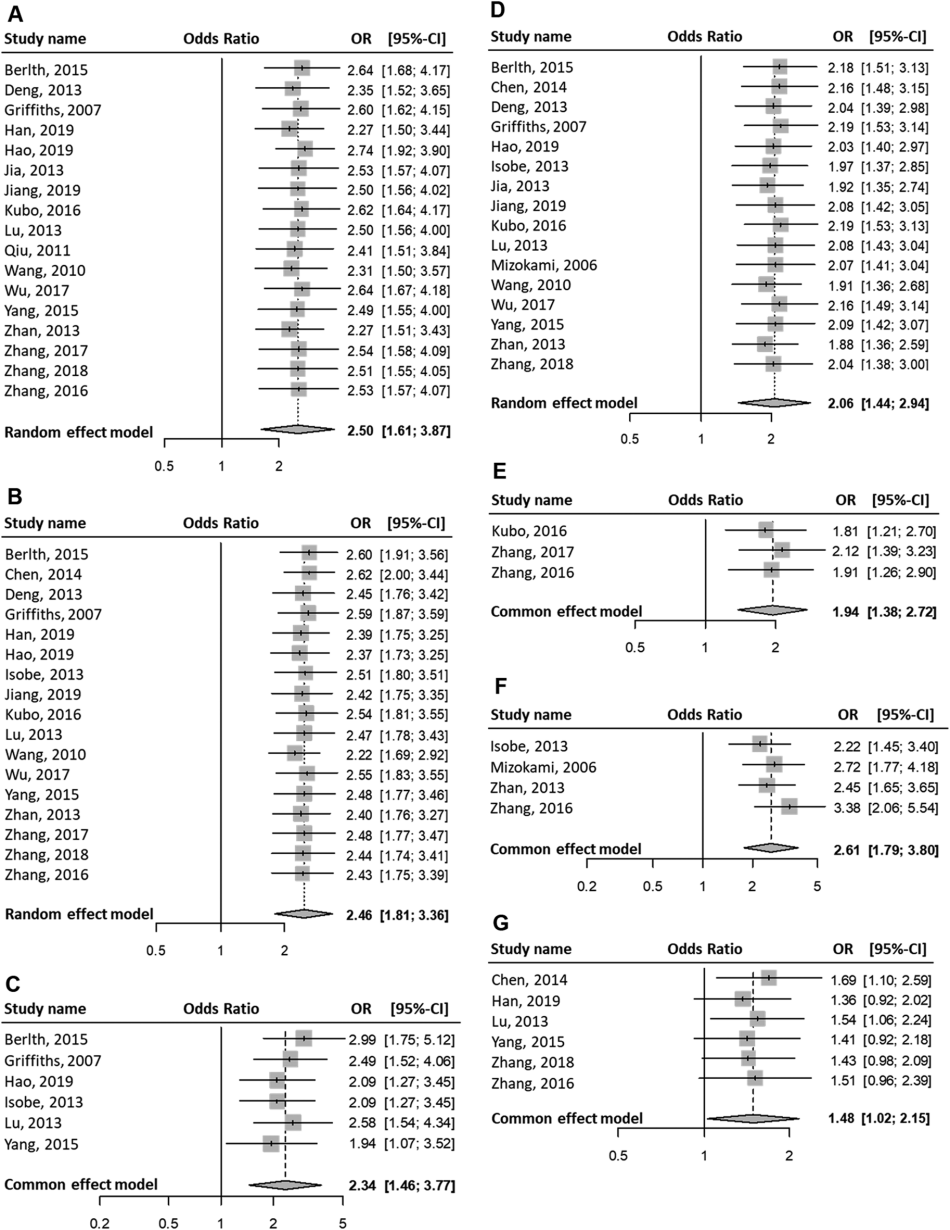

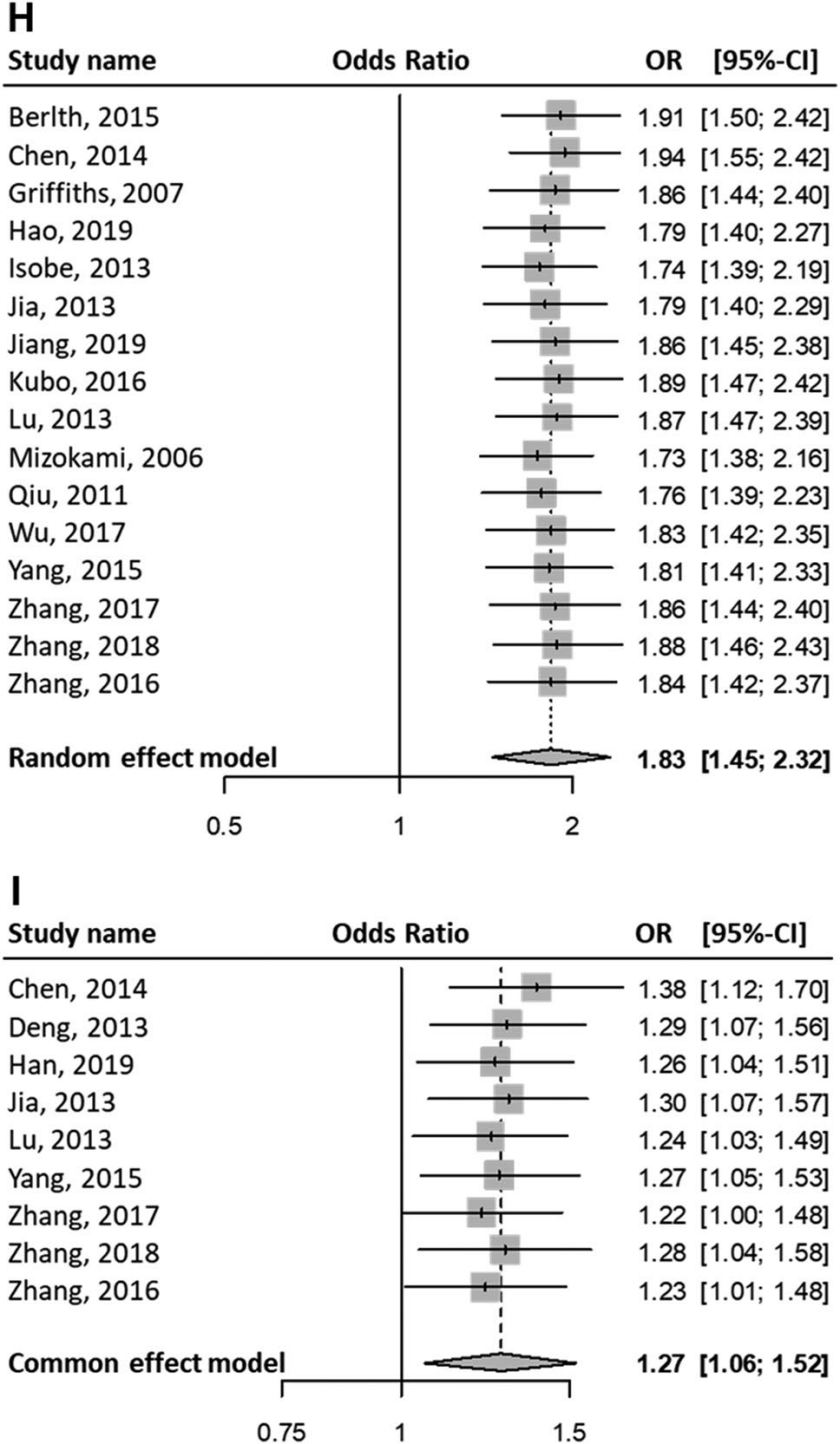
Sensitivity analyses. (**A**) TNM stages III–IV vs. I–II. (**B**) T stages T3–T4 vs. T1–T2. (**C**) M stages M1 vs. M0. (**D**) N stages N1–N3 vs. N0. (**E**) Positive vs. negative vascular invasion. (**F**) High (positive) vs. low (negative) VEGF expression. (**G**) Borrmann types 4–5 vs. 1–3. (**H**) Undifferentiated vs. differentiated differentiation statuses. (**I**) Tumor sizes ≥ 5 cm vs. < 5 cm.

The ORs were consistent when we performed sensitivity analysis of statistically significant clinicopathological characteristics (Fig. 3). Therefore, the sensitivity analysis for significant clinicopathological characteristics confirmed the robustness of the results.

**Figure 4:**
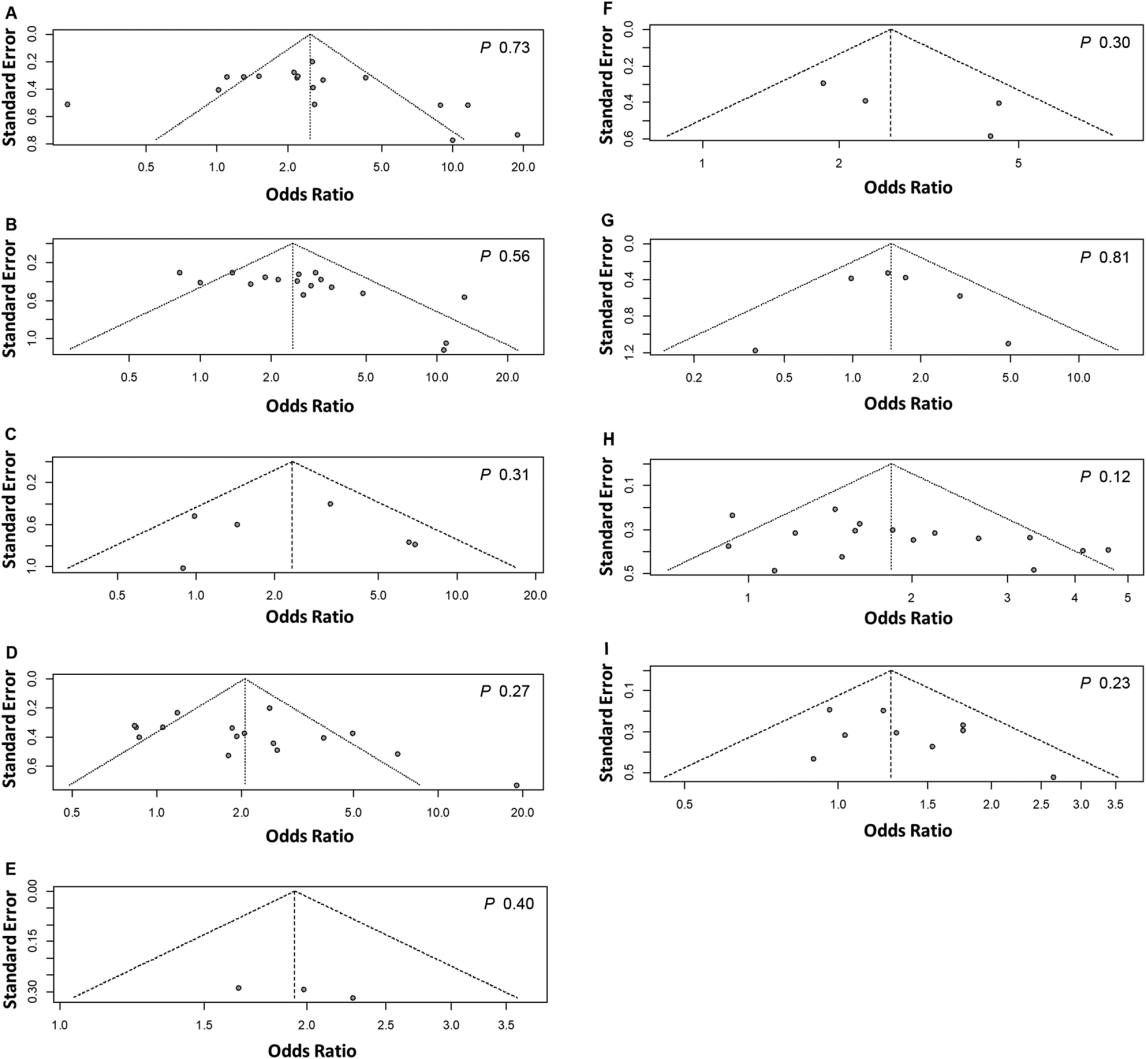
Inspection of funnel plots and publication biases. (**A**) TNM stages III–IV vs. I–II. (**B**) T stages T3–T4 vs. T1–T2. (**C**) M stages M1 vs. M0. (**D**) N stages N1–N3 vs. N0. (**E**) Positive vs. negative vascular invasion. (**F**) High (positive) vs. low (negative) VEGF expression. (**G**) Borrmann types 4–5 vs. 1–3. (**H**) Undifferentiated vs. differentiated differentiation statuses. (**I**) Tumor sizes ≥ 5 cm vs. < 5 cm. P values of Egger’s tests were indicated in the upper right corners of the panels.

The visual assessment of the funnel plots revealed no asymmetry, suggesting the possibility of publication bias (Fig. 4). Egger's tests indicated no statistical significance as well. Overall, the funnel plots and Egger's tests revealed no evidence of publication bias.

### Meta-regression

Age exhibited a significant association with subgroups categorized by TMN stage progression, T stage progression, N stage progression, and differentiation statuses (Supplementary Fig. S1a–d), suggesting age as a source of heterogeneity. Conversely, while sex did not show a significant association with subgroups divided by T stage progression, it displayed a significant association with subgroups categorized by TMN stage progression, N stage progression, and differentiation statuses (Supplementary Fig. S1e–h), indicating sex as a source of heterogeneity in subgroups categorized by TMN stage progression, N stage progression, and differentiation statuses.

## Discussion

The purpose of our study was to determine whether GC patients with high protein levels of HIF1A had more severe clinical features compared with those who did not. We found that the T stage progression, TNM stage progression, lymph node metastasis, differentiated status, M stage progression, Borrmann stage progression, tumor size, vascular invasion, and VEGF protein expression were significantly associated with GC patients with high levels of the HIF1A protein.

Our meta-analysis inspected 20 publications compared with nine studies evaluated in a previous meta-analysis of HIF1A protein expression in GC^23^, enhancing the knowledge of the correlation between HIF1A protein expression and clinical characteristics. Also, this meta-analysis revealed broader correlations between the protein expression and clinical characteristics when compared to the previous meta-analysis^23^.

HIF1A is a functionally important mediator in GC^6^. Activated HIF1A recruits M2-type tumor-associated macrophages (TAMs), aiding chemoresistance in GC^35^. It is also associated with resistance to anoikis—cell death due to the detachment from the extracellular matrix—in GC^36^. Inhibition of HIF1A in GC induced anoikis via integrin-5^36^.

HIF1A is also a mediator of EMT^6^ which promotes tumor progression in the advanced stages^37^. In this meta-analysis, the association between cancer stage progression and positive HIF1A protein expression may support HIF1A-related EMT. EMT affects cellular morphology changes mostly by inhibiting genes involved in differentiation^37^. This aligns with our findings linking HIF1A protein expression to poorly differentiated GC.

Additionally, EMT contributes to the progression of the Lauren diffuse subtype in GC^38^. Considering the involvement of HIF1A in EMT^37^, the association between the diffuse subtype and positive HIF1A expression could be predicted, but the association was not statistically significant in our meta-analysis. A previous meta-analysis comprising nine studies on HIF1A and clinicopathological features in GC^10^ reported that positive HIF1A protein level was associated with TNM stage progression, differentiated GC cell status, T stage progression, vascular invasion, and lymph node metastasis. These results were consistent with our meta-analysis results. New findings of our meta-analysis indicated the statistical associations between positive HIF1A expression and other clinicopathological features, including Borrmann stage progression, positive VEGF protein expression, and tumor sizes.

Based on the functional roles of HIF1A and the results of our meta-analysis, HIF1A signaling is essential for GC progression, and the protein is a potential biomarker. Meta-analyses can provide information on HIF1A protein expression for patient classification. HIF1A is associated with PD-L1, a target of immune checkpoint inhibitors in cancer^39^. In the hypoxic tumor microenvironment, HIF1A binds to the PD-L1 promoter region, upregulating PD-L1 in myeloid-derived suppressor cells and tumor cells^40^.

We believe that this systematic review and meta-analysis provide a complete picture of the clinical association of HIF1A protein expression with GC. Also, the 3416 patients from the 20 studies offered statistical support for a reliable meta-analysis.

However, this study has some limitations. As we used published papers in our meta-analysis, publication bias is unavoidable, which means statistical heterogeneity is inescapable^41^. Publication bias may be due to different patient sources and IHC scoring schemes for measuring protein expression in the selected papers. Additionally, the HIF1A antibodies used to stain the protein varied (Table 1) and might contribute to publication bias.

## Conclusions

According to our research, high levels of HIF1A protein in GC are associated with T stage progression, TNM stage progression, lymph node metastasis, differentiated status, M stage progression, Borrmann stage progression, tumor size, vascular invasion, and positive VEGF protein expression, providing potential biological indicators for the diagnosis and prognosis of patients with GC. Our findings suggest that prospective, large-scale cohort studies are needed to verify HIF1A protein level as a biomarker candidate for GC development.

### Supplementary Information

Supplementary Table S1.

## Data Availability

The original contributions presented in the study are included in the article/supplementary material, further inquiries can be directed to the corresponding author.
