# Supplementary material for: HIF1A protein expression is correlated with clinical features in gastric cancer: an updated systematic review and meta-analysis
Source: Sci Rep. 2024 Jun 14;14:13736. doi: 10.1038/s41598-024-63019-6 (PMC11178933; doi:10.1038/s41598-024-63019-6)
Supplement: Supplementary file 1 — Supplementary Table S1. [file 41598_2024_63019_MOESM1_ESM.pdf]

Supplementary Information

HIF1A Protein Expression is Correlated with Clinical Features in Gastric Cancer: An Updated Systematic Review and Meta-Analysis

Nam and Lee

Contents

Supplementary Table S1.
Supplementary Figure S1.

Supplementary Table S1. Evaluating evidence-quality for our 20 selected studies using the guidelines of Robinson et al. [1]. ("2A: weak recommendation; limited quality; patient-oriented evidence. B: Systematic review/meta-analysis of lower quality cohort studies with inconsistent results that may vary depending on circumstances or patients or societal values; retrospective cohort studies; case-control study [1].")

Authors (study names)	Grade of Recommendation	Quality of Evidence
Berlth et al. [2] (Berlth, 2015)	2A	B
Hao et al. [3] (Hao, 2019)	2A	B
Isobe et al. [4] (Isobe, 2013)	2A	B
Jia et al. [5] (Jia, 2013)	2A	B
Mizokami et al. [6] (Mizokami, 2006)	2A	B
Wu et al. [7] (Wu, 2017)	2A	B
Zhang et al. [8] (Zhang, 2016)	2A	B
Lu et al. [9] (Lu, 2013)	2A	B
Zhan et al. [10] (Zhan, 2013)	2A	B
Yang et al. [11] (Yang, 2015)	2A	B
Qiu et al. [12] (Qiu, 2011)	2A	B
Wang et al. [13] (Wang, 2010)	2A	B
Chen et al. [14] (Chen, 2014)	2A	B
Zhang et al. [15] (Zhang, 2017)	2A	B
Griffiths et al. [16] (Griffiths, 2007)	2A	B
Deng et al [17] (Deng, 2013)	2A	B
Han et al. [18] (Han, 2019)	2A	B
Zhang et al. [19] (Zhang, 2018)	2A	B
Kubo et al. [20] (Kubo, 2016)	2A	B
Jiang et al. [21] (Jiang, 2019)	2A	B

Supplementary Figure S1. The mixed-effects meta-regression model analysis of the outcomes (i.e., odds ratio) on covariates. In the meta-regression model, we obtained *P* values for testing the slope (under the null hypothesis of no linearity between covariates and effect sizes) and *P* values for testing heterogeneity. In the bubble plots, a study was represented by a circle in coordinates (observed effect size, OR, by a covariate) for the study. The size of a circle is proportional to that study's covariate weight assigned by the meta-regression. Dot lines are 95% confidence intervals. The solid line indicates the fitted values.

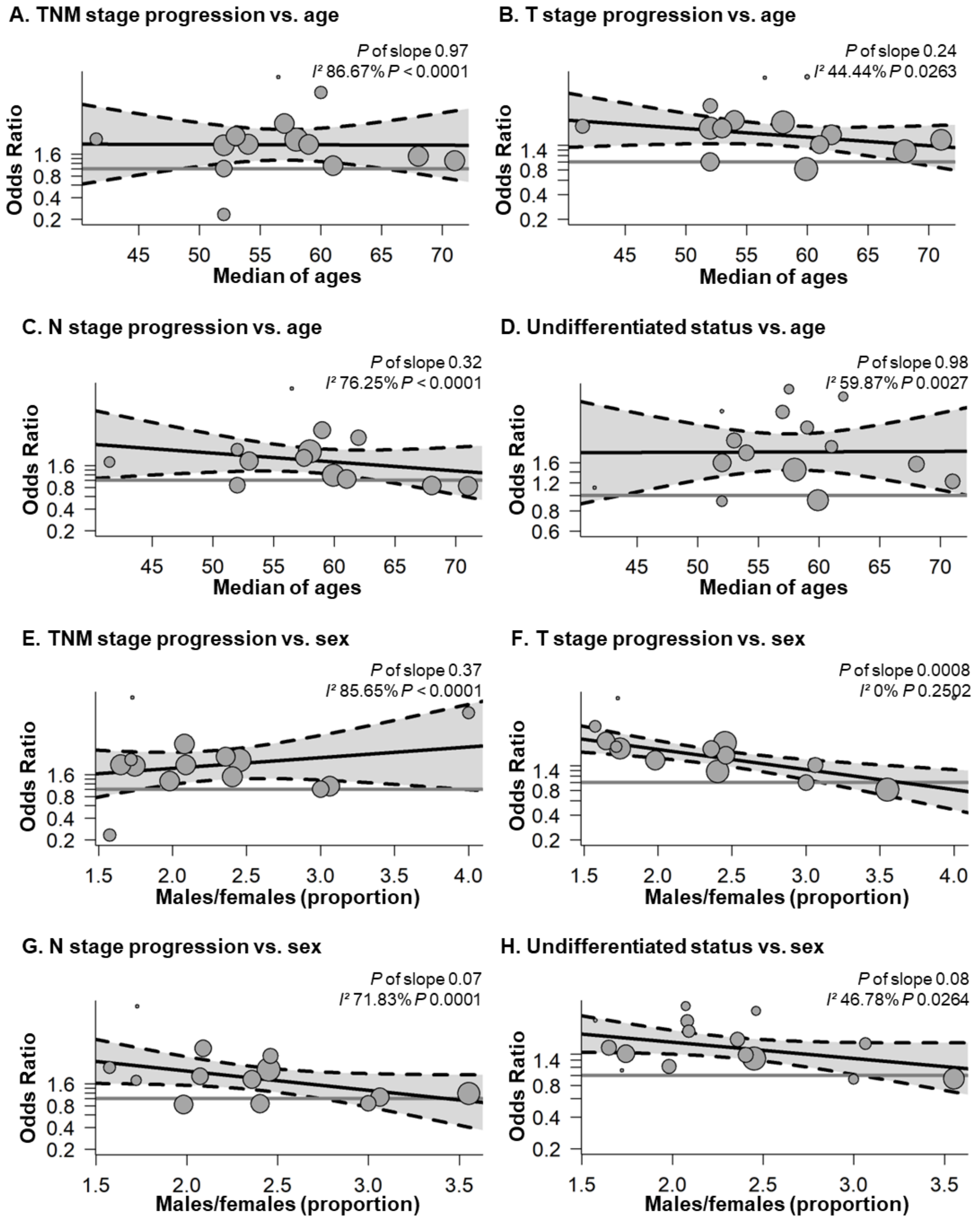

Supplementary References

1 Robinson, J. K., Dellavalle, R. P., Bigby, M. & Callen, J. P. Systematic reviews: grading recommendations and evidence quality. *Arch Dermatol* **144**, 97-99, doi:10.1001/archdermatol.2007.28 (2008).

2 Berlth, F. *et al.* Both GLUT-1 and GLUT-14 are Independent Prognostic Factors in Gastric Adenocarcinoma. *Ann Surg Oncol* **22 Suppl 3**, S822-831, doi:10.1245/s10434-015-4730-x (2015).

3 Hao, L. S. *et al.* Correlation and expression analysis of hypoxia-inducible factor 1alpha, glucose transporter 1 and lactate dehydrogenase 5 in human gastric cancer. *Oncol Lett* **18**, 1431-1441, doi:10.3892/ol.2019.10457 (2019).

4 Isobe, T. *et al.* Clinicopathological significance of hypoxia-inducible factor-1 alpha (HIF-1alpha) expression in gastric cancer. *Int J Clin Oncol* **18**, 293-304, doi:10.1007/s10147-012-0378-8 (2013).

5 Jia, Y. F. *et al.* Differentiated embryonic chondrocyte-expressed gene 1 is associated with hypoxia-inducible factor 1alpha and Ki67 in human gastric cancer. *Diagn Pathol* **8**, 37, doi:10.1186/1746-1596-8-37 (2013).

6 Mizokami, K. *et al.* Clinicopathologic significance of hypoxia-inducible factor 1alpha overexpression in gastric carcinomas. *J Surg Oncol* **94**, 149-154, doi:10.1002/jso.20568 (2006).

7 Wu, Y. *et al.* Down regulation of RNA binding motif, single-stranded interacting protein 3, along with up regulation of nuclear HIF1A correlates with poor prognosis in patients with gastric cancer. *Oncotarget* **8**, 1262-1277, doi:10.18632/oncotarget.13605 (2017).

8 Zhang, W.-J. *et al.* Elevated expression of hypoxia inducible factor-1 alpha is correlates to recurrence and poor outcome in gastric cancer. *Int J Clin Exp Med* **9**, 7209-7216 (2016).

9 Lu, X. X. *et al.* Expression and clinical significance of CD73 and hypoxia-inducible factor-1alpha in gastric carcinoma. *World J Gastroenterol* **19**, 1912-1918, doi:10.3748/wjg.v19.i12.1912 (2013).

10 Zhan, H. *et al.* Expression of Rac1, HIF-1alpha, and VEGF in gastric carcinoma: correlation with angiogenesis and prognosis. *Onkologie* **36**, 102-107, doi:10.1159/000348525 (2013).

11 Yang, Y. *et al.* Expression of RAP1B is associated with poor prognosis and promotes an aggressive phenotype in gastric cancer. *Oncol Rep* **34**, 2385-2394, doi:10.3892/or.2015.4234 (2015).

12 Qiu, M. Z. *et al.* Expressions of hypoxia-inducible factor-1alpha and hexokinase-II in gastric adenocarcinoma: the impact on prognosis and correlation to clinicopathologic features. *Tumour Biol* **32**, 159-166, doi:10.1007/s13277-010-0109-6 (2011).

13 Wang, Y. *et al.* HIF-1alpha and HIF-2alpha correlate with migration and invasion in gastric cancer. *Cancer Biol Ther* **10**, 376-382, doi:10.4161/cbt.10.4.12441 (2010).

14 Chen, L. *et al.* HIF-1 alpha overexpression correlates with poor overall survival and disease-free survival in gastric cancer patients post-gastrectomy. *PLoS One* **9**, e90678, doi:10.1371/journal.pone.0090678 (2014).

15 Zhang, W. J. *et al.* Hypoxia-inducible factor-1 alpha Correlates with Tumor-Associated Macrophages Infiltration, Influences Survival of Gastric Cancer Patients. *J Cancer* **8**, 1818-1825, doi:10.7150/jca.19057 (2017).

16 Griffiths, E. A. *et al.* Hypoxia-inducible factor-1alpha expression in the gastric carcinogenesis sequence and its prognostic role in gastric and gastro-oesophageal adenocarcinomas. *Br J Cancer* **96**, 95-103, doi:10.1038/sj.bjc.6603524 (2007).

17 Deng, B. *et al.* Intratumor hypoxia promotes immune tolerance by inducing regulatory T cells via TGF-beta1 in gastric cancer. *PLoS One* **8**, e63777, doi:10.1371/journal.pone.0063777 (2013).

18 Han, Y. L. *et al.* Lysyl oxidase and hypoxia-inducible factor 1alpha: biomarkers of gastric cancer. *World J Gastroenterol* **25**, 1828-1839, doi:10.3748/wjg.v25.i15.1828 (2019).

19 Zhang, J. *et al.* Prognostic value of hypoxia-inducible factor-1 alpha and prolyl 4-hydroxylase beta polypeptide overexpression in gastric cancer. *World J Gastroenterol* **24**, 2381-2391, doi:10.3748/wjg.v24.i22.2381 (2018).

20 Kubo, H. *et al.* Regulation and clinical significance of the hypoxia-induced expression of ANGPTL4 in gastric cancer. *Oncol Lett* **11**, 1026-1034, doi:10.3892/ol.2015.4011 (2016).

21 Jiang, X. *et al.* The correlation between NEDD4L and HIF-1alpha levels as a gastric cancer prognostic marker. *Int J Med Sci* **16**, 1517-1524, doi:10.7150/ijms.34646 (2019).
